# Deletion of Cyclophilin D Impairs β-Oxidation and Promotes Glucose Metabolism

**DOI:** 10.1038/srep15981

**Published:** 2015-10-30

**Authors:** Michele Tavecchio, Sofia Lisanti, Michael J. Bennett, Lucia R. Languino, Dario C. Altieri

**Affiliations:** 1 Prostate Cancer Discovery and Development Program, Tumor Microenvironment and Metastasis Program, The Wistar Institute, Philadelphia, PA 19104; 2 Michael Palmieri Metabolic Laboratory, Children’s Hospital of Philadelphia and Department of Pathology and Laboratory Medicine, University of Pennsylvania Perelman School of Medicine, Philadelphia, PA 19104; 3 Department of Cancer Biology, Kimmel Cancer Center, Thomas Jefferson University, Philadelphia, PA 19107.

## Abstract

Cyclophilin D (CypD) is a mitochondrial matrix protein implicated in cell death, but a potential role in bioenergetics is not understood. Here, we show that loss or depletion of CypD in cell lines and mice induces defects in mitochondrial bioenergetics due to impaired fatty acid β-oxidation. In turn, CypD loss triggers a global compensatory shift towards glycolysis, with transcriptional upregulation of effectors of glucose metabolism, increased glucose consumption and higher ATP production. *In vivo*, the glycolytic shift secondary to CypD deletion is associated with expansion of insulin-producing β-cells, mild hyperinsulinemia, improved glucose tolerance, and resistance to high fat diet-induced liver damage and weight gain. Therefore, CypD is a novel regulator of mitochondrial bioenergetics, and unexpectedly controls glucose homeostasis, *in vivo*.

In addition to providing cellular energy^1^, mitochondria play a key role in the initiation of programmed cell death, or apoptosis^2^. Many of the molecular requirements of this response, including the participation of Bcl-2 family proteins^3^, and stepwise loss of organelle integrity^4^, have been elucidated in detail, as well as their impact on disease^5^. However, other aspects of this process are less well-defined, in particular the function of a mitochondrial permeability transition pore (PTP)^6^, whose increased conductance in response to cell death *stimuli* causes swelling of the matrix, uncoupling of the electron transport chain (ETC), and release of apoptogenic proteins, notably cytochrome c^7^, in the cytosol. In this context, the mitochondrial matrix peptidyl-prolyl *cis,trans* isomerase, Cyclophilin D (CypD) has long been recognized as a constituent of the PTP^8^, but the involvement of other organelle proteins in pore formation has been debated^9^,^10^, and the impact of this pathway on necrotic *versus* apoptotic cell death has remained elusive^11^.

In addition, there is evidence that CypD may have broader role(s) in cellular homeostasis beyond PTP function. Largely inferred from the phenotype(s) of CypD deletion or silencing, this may involve mitochondria-to-nuclei retrograde signaling in cell proliferation and migration^12^, regulation of mitochondrial DNA transcription and cellular oxygen consumption^13^,^14^, modulation of macrophage inflammatory responses^15^, and metabolic adaptation, *in vivo*^16^,^17^. In particular, how CypD may affect metabolism, including glucose homeostasis, has been controversial in the literature, with evidence suggesting that CypD deficiency is associated with insulin resistance^17^ or, conversely, protects against insulin resistance, *in vivo*^18^. Elucidating a role of CypD in metabolism is significant, as pharmacologic inhibition of CypD protects against tissue damage during myocardial^19^, or cerebral^20^ ischemia-reperfusion injury, as well as neuronal loss^21^, validating this pathway as a therapeutic target in heart disease^22^,^23^, neurodegeneration^24^ and possibly metabolic imbalance.

In this study, we explored a role of CypD in metabolism, and its potential impact on glucose homeostasis and high fat diet-associated tissue damage.

## Results

### CypD regulation of mitochondrial bioenergetics

**Figure 1:**
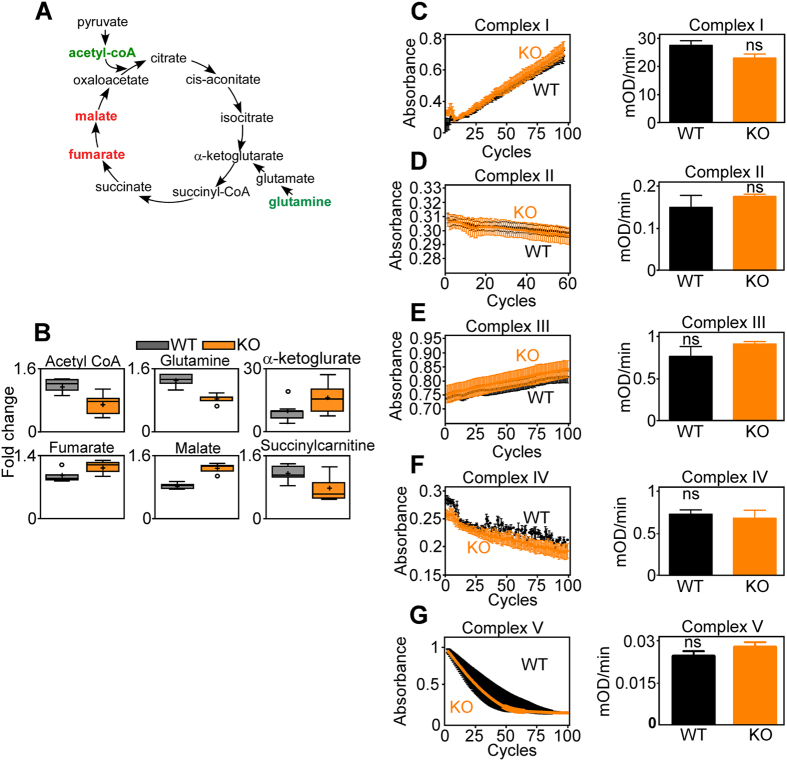
CypD deletion affects TCA cycle but not oxidative phosphorylation. (**A,B**) Wild type (WT) or CypD KO MEFs were analyzed in a global metabolomics screen (n = 5), and changes in expression of metabolites implicated in TCA cycle are schematically represented (**A**) and quantified (**B**). Cellular extracts were used and values were normalized on protein content. For box plots in (**B**), relative metabolite abundance is represented. The limit of upper and lower quartiles, median values (*straight line*), and maximum and minimum distribution are shown. *Cross*, mean value; *circle*, extreme data point. Only significant changes (p < 0.05) are shown. (**C–G**) Mitochondria or mitochondrial extracts (see Material and Methods section) isolated from primary hepatocytes of WT or CypD KO mice (one out of three independent experiments is shown) were analyzed for activity of oxidative phosphorylation Complex I (**C**), Complex II (**D**), Complex III (**E**), Complex IV (**F**) and Complex V (**G**). Tracings (*left*) correspond to individual activity reactions. Bar graphs (*right*), individual mitochondrial complex activity normalized to citrate synthase activity, n = 3.

We began this study by profiling the metabolome of wild type (WT) or CypD knockout (KO) mouse embryonic fibroblasts (MEFs), characterized in recent studies^12^. Deletion of CypD was accompanied by multiple defects in mitochondrial TCA cycle intermediates (Fig. 1A). These included reduced acetyl CoA levels, accumulation of malate and fumarate, depletion of glutamine with concomitant elevation of α-ketoglutarate, potentially suggestive of compensatory glutaminolysis, and decreased levels of the byproduct, succinylcarnitine (Fig. 1B). Despite the defects in cellular respiration, individual mitochondrial oxidative phosphorylation Complex I (Fig. 1C), Complex II (Fig. 1D), Complex III (Fig. 1E), Complex IV (Fig. 1F) and Complex V (Fig. 1G) had comparable activity in WT or CypD KO MEFs.

**Figure 2:**
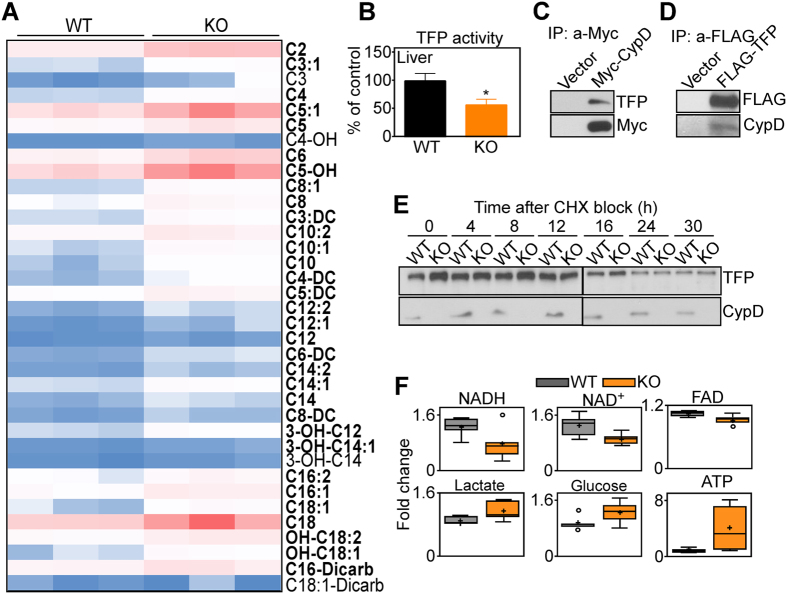
Involvement of CypD in mitochondrial β-oxidation. (**A**) Heatmap of protein-normalized quantification of acylcarnitine levels in WT or CypD KO MEFs in the presence of 5 mM glucose. The figure represents the average of three independent experiments, each run in triplicate. The individual acylcarnitines are indicated. Bold names indicate statistically significant differences. Darkest blue indicates lowest level, darkest red indicates highest level. (**B**) TFP activity was assessed in WT and CypD KO mitochondrial liver extracts. The panel shows a representative experiment out of two, each run in triplicate. (**C,D**) CypD WT MEFs were transfected with a Myc-CypD (**C**) or FLAG-TFP (**D**) cDNA, immunoprecipitated with an antibody to Myc or FLAG, respectively, and immune complexes were analyzed by Western blotting. The two panels are from two different experiments, therefore are from two different electrophoresis runs. No image manipulations were performed and images were cropped only to bring close the two proteins that have a quite different molecular weight. Full gels images are in Supplementary informations file, Fig. 5. The panels show a representative experiment out of two. (**E**) WT or CypD KO MEFs were treated with cycloheximide (CHX), harvested at the indicated time points, and analyzed by Western blotting. The panel shows a representative experiment out of two. The right and left panels are from the same experiment, samples were loaded on two different gels as many time points were performed and could not be allocated on one gel. The development was performed together on the same films. No image manipulations were performed and images were cropped only to bring close the two proteins that have a quite different molecular weight and to indicate the two gels. Full gels images are in Supplementary informations file, Fig. 6. (**F**) Wild type (WT) or CypD KO MEFs were analyzed in a global metabolomics screen (n = 5). Cellular extracts were used and values were normalized on protein content. Relative metabolite abundance is represented. The limit of upper and lower quartiles, median values (*straight line*), and maximum and minimum distribution are shown. *Cross*, mean value; *circle*, extreme data point. Only significant changes (p < 0.05) are shown.

In addition, deletion of CypD was accompanied by an accumulation of acylcarnitine species, consistent with defective mitochondrial fatty acid β-oxidation (Fig. 2A). One of the key regulators of mitochondrial fatty acid metabolism is the α/β octameric trifunctional protein (TFP), which catalyzes the four chain-shortening reactions in β-oxidation, including 3-hydroxyacyl-CoA dehydrogenase (HADH)^25^. Accordingly, TFP activity was significantly reduced in liver extracts from CypD KO mice, compared to WT littermates (Fig. 2B). In addition, recombinant CypD immunoprecipitated from MEF extracts contained endogenous TFP (Fig. 2C), and, reciprocally, FLAG-TFP immune complexes contained co-associated CypD (Fig. 2D). In cycloheximide block experiments, deletion of CypD did not significantly affect TFP stability and turnover, compared to WT cultures (Fig. 2E). Consistent with impaired mitochondrial bioenergetics, CypD loss resulted in decreased content of cofactors, NADH, NAD^+^ and FAD (Fig. 2F). This response was accompanied by increased levels of lactate and glucose, suggestive of heightened glucose metabolism, and overall higher ATP production in CypD KO MEFs, compared to WT cultures (Fig. 2F). Consistent with metabolic shift, CypD KO hepatocytes exhibited global transcriptional changes in bioenergetics effectors, with increased mRNA expression of genes involved in glucose metabolism (Pdk), gluconeogenesis (Fbp1, Pck1) and glycolysis (AldoB, Eno2, Galm, Gapdh, HK3, Pgam2) (Supplementary Figure 1A–C). In contrast, TCA gene products were largely unchanged between WT and CypD KO MEFs (Supplementary Figure 1D).

### CypD deletion induces enhanced glycolysis

**Figure 3:**
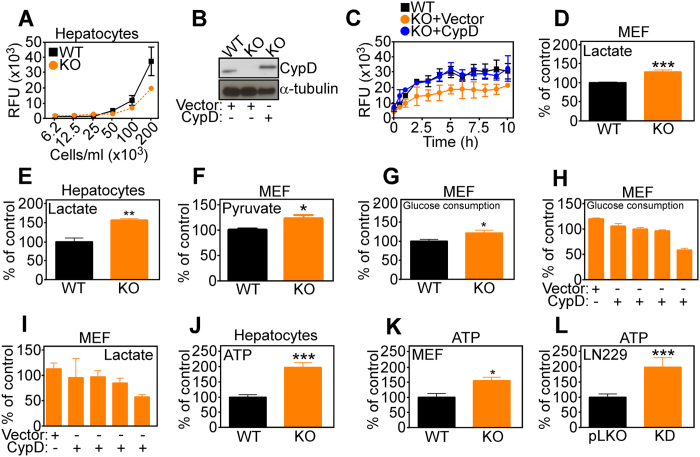
Glucose metabolism switch secondary to CypD deficiency. (**A**) Primary hepatocytes isolated from WT or CypD KO mice were analyzed for oxygen consumption at the indicated cell densities. The panel shows a representative experiment out of two, each run in triplicates. RFU = Relative Fluorescence Unit. (**B,C**) CypD KO MEFs were reconstituted with vector or a CypD cDNA and analyzed by Western blotting (**B**) and changes in O_2_ consumption at increasing time intervals (**C**) RFU = Relative Fluorescence Unit. WT MEFs were analyzed as control. The panel shows a representative experiment out of two, each run in triplicates. The two panels in (**B**) are from the same experiment, therefore are from the same electrophoresis run. No image manipulations were performed and images were cropped only to bring close the two proteins that have a quite different molecular weight. Full gels images are in Supplementary informations file, Fig. 7. (**D,E**) MEFs (**D**) or primary hepatocytes (**E**) isolated from WT or CypD KO mice were analyzed for lactate production. The panel shows a representative experiment out of two, each run in triplicates. (**F**) WT or CypD KO MEFs were analyzed for pyruvate production. The panel shows a representative experiment out of two, each run in triplicates. (**G**) MEFs were analyzed for glucose consumption. The panel shows a representative experiment out of two, each run in triplicates. (**H,I**) CypD KO MEFs were reconstituted with vector or increasing concentrations of a CypD cDNA (0.1, 0.25, 0.5 and 1 μg/ml) and analyzed for glucose consumption (**H**) or lactate production (**I**). Mean ± SD of a representative experiment. (**J–L**) Primary hepatocytes (**J**) or MEFs (**K**) isolated from WT or CypD KO mice were analyzed for ATP production. The panel shows a representative experiment out of two, each run in triplicates. (**L**) Glioblastoma LN229 cells stably transfected with control shRNA or CypD-directed shRNA were analyzed for ATP production. The panel shows a representative experiment out of two, each run in triplicates.

Consistent with defective mitochondrial β-oxidation, CypD KO hepatocytes exhibited significantly reduced oxygen consumption, compared to WT cultures (Fig. 3A). To establish a role of CypD in this response, we next carried out reconstitution experiments in CypD KO MEF. In these experiments, reconstitution of CypD KO cells with CypD cDNA (Fig. 3B) restored O_2_ consumption indistinguishably from WT cells, whereas a control plasmid was ineffective (Fig. 3C). To independently validate our findings, we next silenced the expression of Hadha, one of the two subunits of the TFP, and looked at potential changes in cellular respiration. In these experiments, siRNA silencing of Hadha in WT MEF significantly inhibited oxygen consumption to levels observed in CypD KO cells (Supplementary Figure 4A). Together with the results above (Fig. 1C–G), these data support the model that CypD deletion impairs cellular respiration by decreasing β-oxidation, without directly affecting oxidative phosphorylation. To confirm the sensitivity of the approach, we next quantified oxygen consumption in lung adenocarcinoma A549 ρ^o^ cells, which have been depleted of mitochondrial DNA and are thus respiration-incompetent. Compared to parental A549 cells, which had robust oxygen consumption, A549 ρ^o^ cells were ineffective (Supplementary Figure 4B), thus validating the experimental protocol used. In agreement with the data of metabolomics profiling, CypD deletion in MEFs or primary hepatocytes was associated with increased production of lactate (Fig. 3D,E), as well as pyruvate (Fig. 3F). This was associated with heightened glucose consumption (Fig. 3G). We next examined the specificity of these responses, and reconstitution of CypD KO cells with CypD cDNA normalized glucose consumption (Fig. 3H), as well as lactate production (Fig. 3I), compared to control plasmid. Together, the increased glucose metabolism associated with CypD deletion resulted in higher levels of ATP production in hepatocytes (Fig. 3J) or MEFs (Fig. 3K). In addition, stable shRNA knockdown of CypD in glioblastoma LN229 cells also resulted in increased ATP production, compared to control cultures transfected with non-targeting shRNA (Fig. 3L).

### CypD deletion heightens glucose metabolism, *in vivo*

**Figure 4:**
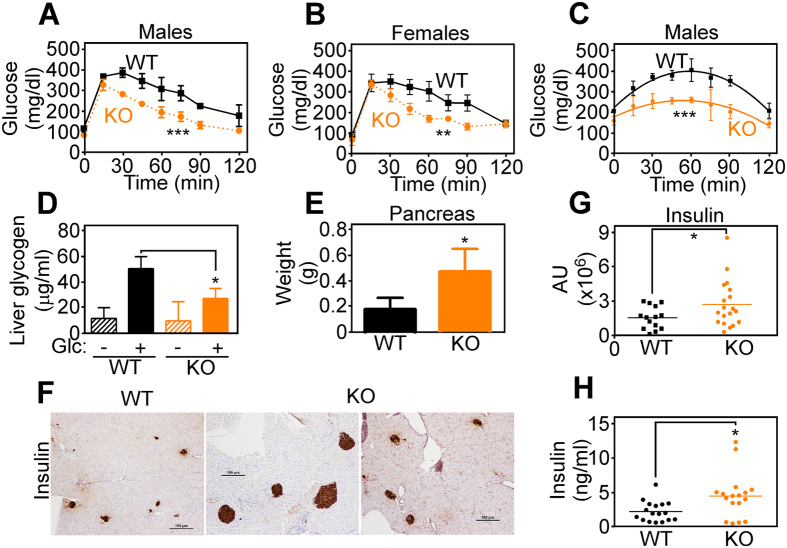
*In vivo* glucose metabolism is enhanced in CypD KO mice. (**A,B**) WT or CypD KO male (**A**) or female (**B**) mice were challenged in a glucose tolerance test with i.p. injection of glucose (2 mg/g), and analyzed for blood glucose levels at the indicated time intervals The panel shows a representative experiment out of two, each run in triplicates (**C**) WT or CypD KO mice were challenged with i.p. injection of pyruvate (2 mg/g) and analyzed for blood glucose levels at the indicated time intervals. The panel shows a representative experiment out of two, each run in triplicates. (**D**) WT or CypD KO mice were injected with glucose and analyzed for changes in liver glycogen after 30 min. Three animals per group, each measured three times. (**E**) Pancreas tissues were isolated from 3-mo old WT or CypD KO mice and weighed. n = 5. (**F**,**G**) Pancreas sections from WT or CypD KO mice were stained with an antibody to insulin by immunohistochemistry (**F**), and labeled areas were quantified and multiplied by relative intensity (**G**). Magnification, x20; data represent 14 different fields out of 5 WT animals, compared to 19 fields out of 5 KO animals. (**H**) Blood samples collected from WT or CypD KO mice were analyzed for insulin levels. Each point corresponds to an individual animal. n = 17, each measured in triplicate.

Next, we looked at the implications of glucose metabolism in KO *versus* WT CypD mice, *in vivo*. In glucose tolerance tests, both male and female CypD KO mice exhibited faster normalization of glycemia, compared to WT CypD littermates (Fig. 4A,B and supplementary Figure 2A,B). Similar results were obtained in pyruvate tolerance tests, with more rapid normalization of glycemia in CypD KO mice, compared to WT animals after systemic administration of pyruvate (Fig. 4C and supplementary Figure 2C). Under these conditions, livers from CypD KO mice exhibited reduced glycogen content following a bolus of glucose, as compared with CypD WT animals (Fig. 4D). A potential mechanism for the improved glucose tolerance observed in CypD KO mice was next investigated. Accordingly, pancreas tissues collected from CypD KO mice exhibited increased weight, (Fig. 4E), and higher number of insulin-producing islet β-cells, compared to WT animals (Fig. 4F,G). Consistent with these data, CypD KO mice showed higher levels of circulating plasma insulin than WT littermates (Fig. 4H).

**Figure 5:**
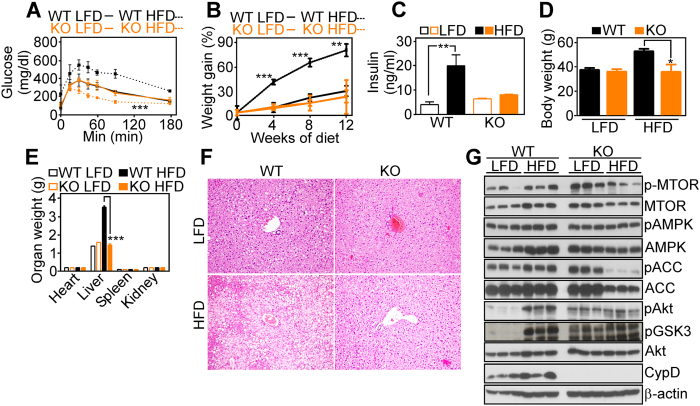
*In vivo* protective effects induced by CypD deletion. (**A,B**) WT or CypD KO mice were maintained on low-fat diet (LFD) or high-fat diet (HFD) for 12 weeks, and animals in the various groups were analyzed for glucose tolerance after i.p. injection of glucose (2 mg/g) (**A**) or changes in overall body weight (compared to LFD animals at day 0, (**B**). The panels show one out of two representative experiments, each run with four animals per group. (**C-E**) The experimental conditions are as in (**A**) and mice in the various groups were quantified for insulinemia (**C**), overall body weight (**D**) or weight of the indicated organs (**E**). (**F**) Liver sections from representative mice in the various groups as in (**B**) were analyzed histologically by hematoxylin/eosin staining. Magnification, x20. Livers from all the animals were analyzed and a representative one from each group is shown. (**G**) Total liver extracts from WT or CypD KO mice exposed to LFD or HFD were analyzed by Western blotting. Three different mice were used for each condition. The panel is an entire experiment, therefore from a single electrophoresis run. No image manipulations were performed and images were cropped only to bring close the analyzed proteins. Different exposures were used for the different proteins, due to their different abudancies and the different antibodies avidity. Full gels images are in Supplementary informations file, Fig. 8.

The potential implications of glucose metabolism in CypD KO mice were further investigated. Exposure of WT mice to a 12-week course of high-fat diet (HFD) resulted in glucose intolerance in response to systemic administration of a glucose bolus, while KO animals were protected and cleared glucose even faster than their LFD-fed counterpart (Fig. 5A and supplementary Figure 2D). In addition, CypD WT mice exposed to HFD exhibited hyperinsulinemia, while KO mice were protected (Fig. 5C). Moreover, WT animals exposed to HFD exhibited progressive weight gain (Fig. 5B,D), and increase in liver weight (Fig. 5E). Histologically, this was associated with extensive tissue damage and lipid accumulation (Fig. 5F). In contrast, CypD KO mice treated with HFD under the same conditions normalized blood glucose levels within 90 minutes of bolus injection (Fig. 5A), and showed no weight gain (Fig. 5B,D). By the end of treatment, HFD-fed CypD KO mice showed no changes in liver weight compared to control animals fed normal diet (Fig. 5E), and had no histologic evidence of lipid accumulation in the liver (Fig. 5F and Supplementary Figure 3). Other organs, including heart, spleen and kidneys showed no weight difference in WT or CypD KO mice fed low- or high-fat diet (Fig. 5E). In analysis of liver extracts, WT mice exposed to HFD revealed increased phosphorylation of kinases associated with insulin signaling, including AMPK and its downstream target, ACC, mTOR, and Akt, compared to control animals fed normal diet (Fig. 5G). In contrast, CypD deletion was associated with constitutively higher levels of phosphorylated kinases, quantitatively similar in high- or low-fat diet (Fig. 5G).

## Discussion

In this study, we have identified a novel role of the mitochondrial matrix protein, CypD^8^ in glucose metabolism, insulin production and liver protection in response to high-fat diet, *in vivo*. Mechanistically, this pathway functions as a compensatory response to impaired mitochondrial β-oxidation resulting from CypD deletion or depletion, and involves transcriptional upregulation of glucose regulatory molecules, improved glucose tolerance, expansion of insulin-producing β-cells and mild hyperinsulinemia, *in vivo*.

Although long established as a component of the PTP^8^, and implicated in the execution of cell death^11^, the function(s) of CypD in mitochondrial bioenergetics have remained elusive. Here, a global metabolomics screen uncovered broad defects in mitochondrial TCA and β-oxidation and reduced oxygen consumption in CypD KO MEFs. Different from previous reports, where CypD deletion did not affect cellular respiration^14^, or reduced the levels of oxidative phosphorylation complex subunits^8^, we observed no difference in the activity of individual mitochondrial oxidative phosphorylation complexes in WT or CypD KO liver mitochondria.

Instead, CypD deletion impaired fatty acid oxidation, with accumulation of acylcarnitines, in agreement with previous findings^14^. Mechanistically, a role of CypD in fatty acid metabolism involves a novel association described here between CypD and TFP^25^, a key effector of organelle β-oxidation^26^, whose activity was significantly reduced in CypD knockout cells. Although more work is required to elucidate how an interaction with CypD may affect TFP activity, this pathway does not appear to involve changes in TFP stability and/or folding in mitochondria.

Defective mitochondrial fatty acid metabolism in CypD knockout cells was accompanied by transcriptional upregulation of glycolysis mediators and a general switch towards glucose metabolism, with higher glucose consumption, lactate levels, and ATP production, all adaptive responses secondary to CypD deletion. In turn, the compensatory glucose metabolism associated with CypD deletion resulted in improved glucose tolerance, expansion of insulin-producing islet β cells, and sustained hyperinsulinemia, *in vivo*. The impact of CypD on glucose tolerance, *in vivo*, has been debated. Consistent with the data presented here, metabolic profiling studies of CypD-targeted cells also suggested a switch towards glycolytic metabolism^16^. However, the implications of this response *in vivo* varied greatly, from improved glucose tolerance due to better mitochondrial Ca^2+^ retention capacity, without changes in insulin signaling^18^, to defective insulin signaling, partially corrected by administration of metformin^17^. Here, analysis of liver tissues from CypD KO mice revealed constitutive upregulation of kinase cascades associated with insulin signaling, including phosphorylated Akt, mTOR and AMPK, in a reaction not further modulated by exposure to high-fat diet, *in vivo*.

In this context, metabolic reprogramming and improved glucose tolerance in CypD knockout mice protected against liver damage induced by high-fat diet. In keeping with its role as a regulator of necrotic cell death^11^, deletion of CypD has been previously associated with improved tissue protection in response to ischemia/reperfusion injury^19^, and neurodegenerative insults^20^. In the case of high-fat diet, deletion of CypD has also been mechanistically linked to inhibition of cell death, independently of inflammation^27^, as well as prevention of mouse obesity^28^.

In summary, we have described a novel role of CypD in metabolic switching and glucose bioenergetics, *in vivo*. The ability of CypD to control glucose homeostasis and insulin production *in vivo* (this work) should be considered in therapeutic strategies aimed at targeting CypD for neurodegeneration^24^, ischemia-reperfusion injury^22^,^23^ and possibly hyperglycemia. Indeed, patients with defective fatty acid oxidation due to a mutation in the TFP are maintained on a strict glucose-rich diet^29^ and TFP KO mice exhibit severe hypoglycemia^30^, thus resembling and strengthening our data and confirming CypD as a player in β-oxidation.

The possibility to therapeutically exploit this shift could be used in pathologies where hyperglycemia is an important feature, for example diabetes. Consistent with this hypothesis, trimetazidine, an Hadha inhibitor used for the treatment of *angina pectoris*, increases glucose uptake in rat brain^31^ and has been reported to have beneficial effects in diabetic patients^32^.

## Materials and Methods

### Cell lines

Wild type (WT) or CypD KO mouse embryonic fibroblasts (MEF) were described previously^12^. Glioblastoma LN229 and A549 lung carcinoma cells were obtained from the American Type Culture Collection (ATCC, Manassas, VA), and maintained in culture at 37 °C in a 5% CO_2_ atmosphere as recommended by the supplier. Cell types were grown in DMEM plus antibiotics, 10% FBS, glutamine and different concentrations of glucose.

### Antibodies and reagents

The following antibodies to Trifunctional Protein (TFP) (AbCam, ab54477), CypD (Millipore, AP1035), FLAG, β-actin and α-tubulin (Sigma-Aldrich, A8592, A5541, T9026) were used. All other antibodies were from Cell Signaling Technology (code for pmTOR: 2971, mTOR: 2983, pAMPK: 2535, AMPK: 2603, pACC: 3661, ACC: 3676, pAkt: 9271, Akt: 9272, pGSK3α/β: 8566).

### Metabolomics screen

A global metabolomics screen to examine changes in expression of 301 individual metabolites was carried out by Metabolon, Inc. in WT or CypD KO MEFs, as described previously^33^. Statistically significant changes were used to build a metabolite set enrichment overview using MSEA (Metabolite Set Enrichment Analysis) software.

### β-oxidation measurements

Acylcarnitines measurement in WT or CypD KO MEFs was carried out by flow-injection tandem mass spectrometry adapted from the method of Shen *et al.*^34^. Fifty microliters of cell lysates were placed in a 96 well plate containing stable isotope-labeled internal standards at a final concentration of 0.2 μM (Cambridge Isotope Laboratories, Andover, MA). The lysate was dried under a steady stream of nitrogen, followed by the addition of 50 μL butanolic HCl, sealed and heated for 15 min at 65 °C to form butyl-esters. Samples were then dried down under a steady stream of nitrogen and finally reconstituted in acetonitrile: water (80:20). Five μl of this product were directly injected into a Xevo-TS S tandem mass spectrometer (Waters Corp. Waltham, MA). Data were acquired in the multiple reaction mode where parent compounds of the carnitine-specific fragment of *m/z* 85 were acquired. Acylcarnitines were quantified against the nearest equivalent internal standard by isotope ratio measurement. Finally, results were normalized on the protein content.

### Immunoprecipitation

Mitochondria from transfected MEF were lysed in 50 mM Tris HCl pH 7.4, 150 mM NaCl, 1 mM EDTA, 1% Triton X-100 plus protease and phosphatase inhibitors. Five-hundred μg of proteins were pre-cleared with agarose beads, and incubated with a primary antibody to Myc or FLAG tags for 16 h at 4 °C. After washes, proteins in precipitated pellets were separated by SDS gel electrophoresis, and western blotting was performed.

### Western blotting

Protein lysates were prepared with an extraction buffer containing 150 mM NaCl, 50 mM Tris pH 8.0, 1% Triton X-100, in the presence of protease and phosphatases inhibitors. Twenty μg of proteins were separated on SDS polyacrylamide gels, blotted onto PVDF membrane and incubated with primary antibodies overnight at 4 °C. Incubation with secondary HRP-conjugated antibodies followed, and immunoreactive bands were detected with ECL reagents (GE Healthcare, RPN2106).

### Hydroxyacyl-CoA dehydrogenase (HADHA) activity

Mitochondrial proteins (200 μg) isolated from WT or CypD KO livers were suspended in buffer containing 50 mM MES, 100 mM KH_2_PO_4_, 100 μM dithiothreitol, 100 μM NADH, supplemented with 50 μM 3-oxoacyl-CoA ester and 0.1% Triton X-100, pH 6.1. HADHA activity representative of trifunctional protein (TFP) enzymatic activity was followed by monitoring the decrease in absorbance at 340 nm. TFP activity was calculated dividing the changes in absorbance over time in the linear range of the readings.

### Glucose consumption assay

Glucose concentrations in the media were measured by a standard glucometer (OneTouch Ultra2). Values from samples were subtracted from the blank (medium with no cells) and then normalized on the respective number of cells.

### Lactate production assay

3 μl of culture media were collected 48 hours after cells seeding and were incubated with 50 μl of Assay Reaction Master Mix for 30 min at 22 °C (L-Lactate Assay Kit – Colorimetric Abcam, cat. no. ab65331). Lactate concentration in the various samples were quantified by absorbance at 450 nm and values were normalized on the cell number.

### Pyruvate production assay

A kit (700470) from Cayman Chemical was used. Medium from MEF culture was collected and deproteinized with an equal volume of 0.5 M of metaphosphoric acid. After centrifugation, potassium carbonate was added to the supernatant to neutralize the acid. The supernatant is then diluted 1:1 with the provided buffer. 20 μl of each samples are used and additioned with 50 μl of assay buffer, 50 μl of FAD and thiamine pyrophosphate, 10 μl of fluorometric detector and 20 μl of pyruvate oxidase and horseradish peroxidase. Ex/Em wavelenghts are 530-540/585-595 nm. Values are then normalized on cell number.

### ATP production assay

Different cell types were seeded in white 96-well plates. After 24 or 48 hours, working solution from the kit (eEnzyme, cat. No. CA-A115) was added, and luminescence was measured. Numbers were then normalized on the cell number.

### Oxygen consumption assay

Cells were seeded at different densities in black 96 wells plates with transparent bottom and 24 hours later medium was changed with DMEM with no phenol red (Gibco, 11054-020).

The probe from a kit (Enzo Life Sciences 51045-K100) was added and the cells covered with mineral oil to avoid flux of oxygen with the atmosphere. At different time points, increase in fluorescence was measured at 650 nm, indicating a proportional utilization of oxygen. Numbers were then normalized on the number of cells.

### Cytrate Synthase activity assay

Two μg of mitochondrial preparation were used to evaluate the citrate synthase activity, with a Cytrate Synthase kit (ScienCell, cat#8318). The assay is based on the reaction between 5′, 5′-Dithiobis-nitrobenzoic acid and CoA-SH to form TNB, which exhibits maximum absorbance at 412 nm. The intensity of the absorbance is proportional to the citrate synthase activity.

### Mitochondrial complexes activity assays

Complexes I to IV activities were measured using Abcam kits number 109721, 109908, 109905 and 109911, respectively, according to manufacturer’s instructions. Briefly, mitochondria were lysed (not for complex III measurements) in the provided buffers to obtain a 5 μg/μL protein solution.

For complex I, 50 μg of proteins were loaded in every well of the provided plate for immunocapturing. After washings, the oxidation of NADH (provided by the kit) to NAD^+^ was indirectly followed with the simultaneous reduction of a provided dye. This reduction was directly proportional to an increase in the 450 nm absorbance, which was constantly monitored.

For complex II, 300 μg of proteins were loaded in every well of the provided plate for immunocapturing. After washings, Ubiquinone 2 and Succinate were added and Ubiquinol was produced. This was coupled with the reduction of the provided dye, monitored by a decrease in absorbance at 600 nm.

For complex III, 12 μg of intact mitochondria were loaded in every well of the provided plate. After washings, Cytochrome C was added and its reduction was followed by the increase in absorbance at 550 nm. Rotenone was included in the reaction to inhibit complex I, while KCN was used to avoid the re-oxydation of Cytochrome C by complex IV.

For complex IV, 100 μg of proteins were loaded in every well of the provided plate for immunocapturing. After washings, Cytochrome C was added and its oxidation was followed by the decrease in absorbance at 550 nm.

Complex V activity was evaluated with a kit from Cayman Chemicals (701000). Briefly, freshly isolated mitochondria at 5 μg/μL underwent two freeze-thaw cycles and in the final reaction 1 μg was used in each well of a 96 wells plate. The assay is based on the conversion of ATP to ADP by the complex, the ADP is then used by pyruvate kinase to form pyruvate from phosphoenolpyruvate. Pyruvate, in the presence of lactate dehydrogenase is reduced to lactate and the concomitant reaction oxidizes NADH to NAD^+^. The rate of NADH oxidation was monitored at 340 nm for 30 minutes every 30 seconds at 25 °C.

Complexes activities were calculated dividing the changes in absorbance over time in the linear range of the readings. Finally, the results were normalized on Cytrate Synthase activity.

### Mitochondrial fractionation

Mitochondria were obtained by use of a commercial kit for isolation (Thermo Scientific, 89874), following the manufacturer’s instructions. Briefly, harvested cells were dounce-homogenized and centrifuged at 700 *g* for 10 minutes. Supernatant were then centrifuged at 3000 *g* for 15 minutes, to obtain mitochondria in the pellet. Samples were then snap-frozen in N_2_ liquid and stored at −80 °C until use.

### Hepatocytes isolation

Hepatocytes from 6–8 weeks old mice were isolated with the two-step collagenase perfusion method and cultivated in collagen-coated plates for the experiments. Hepatocytes were seeded at 200000/mL and kept up to 48 hours in M199 media with 10% FBS and 1% Pen/Strep.

### Transfections

Cells were seeded at 2 × 10^4^/ml and 24 hours later transfected with 0.1 μg/mL of control or HADHA-Flag or CypD-Myc plasmid DNA using Lipofectamine.

### Mice

Pure C57/Bl6 CypD KO mice were kindly donated by Dr. Mike A Forte (Vollum Institute, Oregon Health & Sciences University, Portland, Oregon) and were crossed with WT C57/Bl6 (Jackson Lab) to obtain heterozygous animal, that were then used to propagate the colony. All animal procedures were approved by the Institutional Animal Care and Use Committee (IACUC) at The Wistar Institute and experiments were performed in accordance with the relevant guidelines and regulations published in the Wistar Institute Guide for Animal Research.

### Glucose and pyruvate tolerance test

8 weeks old mice were food-withdrawn for 16 hours (water was provided *ad libitum*) and then injected *intra peritoneum* with 2 mg/g of glucose or pyruvate. Glycemia was checked at different time points by a standard glucometer (OneTouch Ultra2). Harvested tissues were immediately snap-frozen in liquid nitrogen and kept at −80 °C until processing.

### Glycogen measurement

Frozen livers were ground and after lysis of red blood cells a commercial kit (Cayman Chemical, cat. #700480) was used, according to manufacturer’s instructions. Briefly, 300 mg of ground tissue was homogenized in assay buffer and diluted ten times. 10 μL were put in every well of a 96 wells plates, and then 50 μL of hydrolysis buffer with amyloglucosidase were added, to convert glycogen to β-D-glucose. Glucose oxidase was then added to convert β-D-glucose to hydrogen peroxide. Horseradish peroxidase was added and H_2_O_2_, in the presence of it, reacted with 10-acetyl-3,7-dihydroxyphenoxazine to produce resorufin, whose fluorescence was measured at Ex/Em of 540/595 nm.

### Insulin measurements

Insulin was measured with an ELISA kit (Millipore EZRMI-13K), according to manufacturer’s protocol, using 10 μl of mouse serum. Briefly, serum from animals was added to a 96 wells plate coated with anti-insulin antibody. After extensive washings a biotynilated anti-insulin antibody was added and subsequently a streptavidin-conjugated horseradish peroxidase was added. After further washings, HRP substrate is added and the increased absorbance at 450 nm is monitored.

### Insulin IHC

FFPE pancreas sections were deparaffinised and antigen retrieval was performed by heat. After blocking, 1/64000 of anti-insulin antibody (Abcam, ab8304) was added for 16 hours at 4 °C. Secondary, HRP-conjugated antibody was added and the reaction was stopped after 2 minutes. After the slides were mounted, pictures were taken and the intensity and area of insulin islets were quantified by ImageJ and multiplied by each other.

### Cholesterol measurements

A kit from Cayman Chemical (10007640) was used. Serum from mice under LFD or HFD regimens was collected and diluted 400 times. Briefly, 50 μL of samples were used and additioned with the same volume of assay cocktail, composed by assay buffer, cholesterol detector, HRP cholesterol oxidase and cholesterol esterase. Samples were excited at 540 nm and fluorescence detected at 595 nm.

### Triglycerides measurements

A kit from Cayman Chemical (10010303) was used. Triglycerides were measured in serum and feces of mice under LFD or HFD regimens. Briefly, 10 μL of each sample were put in each well, then 150 μL of enzyme buffer were added and the absorbance was read at 540 nm.

### Statistical analysis

Data were analyzed using the two-sided unpaired *t*-tests using GraphPad software. Welch’s correction was applied when the two groups had different sample sizes. Data are expressed as mean ±SD from a representative experiment out of three (unless otherwise stated). Replicates numbers are indicated in the figure legends. A p value of <0.05 was considered statistically significant. *p < 0.05; **p < 0.01; ***p < 0.001; ns, not significant.

## Additional Information

**How to cite this article**: Tavecchio, M. *et al.* Deletion of Cyclophilin D Impairs β-Oxidation and Promotes Glucose Metabolism. *Sci. Rep.* **5**, 15981; doi: 10.1038/srep15981 (2015).

## Supplementary Material

Supplementary Information
