# Supplementary material for: Deletion of Cyclophilin D Impairs β-Oxidation and Promotes Glucose Metabolism
Source: Sci Rep. 2015 Oct 30;5:15981. doi: 10.1038/srep15981 (PMC4626838; doi:10.1038/srep15981)
Supplement: Supplementary Information [file srep15981-s1.pdf]

SUPPLEMENTARY INFORMATIONS

DELETION OF CYCLOPHILIN D IMPAIRS β-OXIDATION AND PROMOTES GLUCOSE METABOLISM

Michele Tavecchio[1], Sofia Lisanti[1], Michael J. Bennett[2], Lucia R. Languino[3], and Dario C. Altieri[1]

[1]Prostate Cancer Discovery and Development Program,

Tumor Microenvironment and Metastasis Program, The Wistar Institute, Philadelphia, PA 19104

[2]Michael Palmieri Metabolic Laboratory, Children's Hospital of Philadelphia and Department of Pathology and Laboratory Medicine, University of Pennsylvania Perelman School of Medicine, Philadelphia, PA 19104

[3]Department of Cancer Biology, Kimmel Cancer Center, Thomas Jefferson University, Philadelphia, PA 19107

Keywords: Cyclophilin D, mitochondria, glycolysis, metabolism, insulin resistance

Running title: Cyclophilin D regulation of glucose metabolism

Correspondence: Dario C. Altieri, M.D.

The Wistar Institute Cancer Center,

3601 Spruce Street, Philadelphia, PA 19104

Tel. (215) 495-6970; (215) 495-2638; daltieri@wistar.org

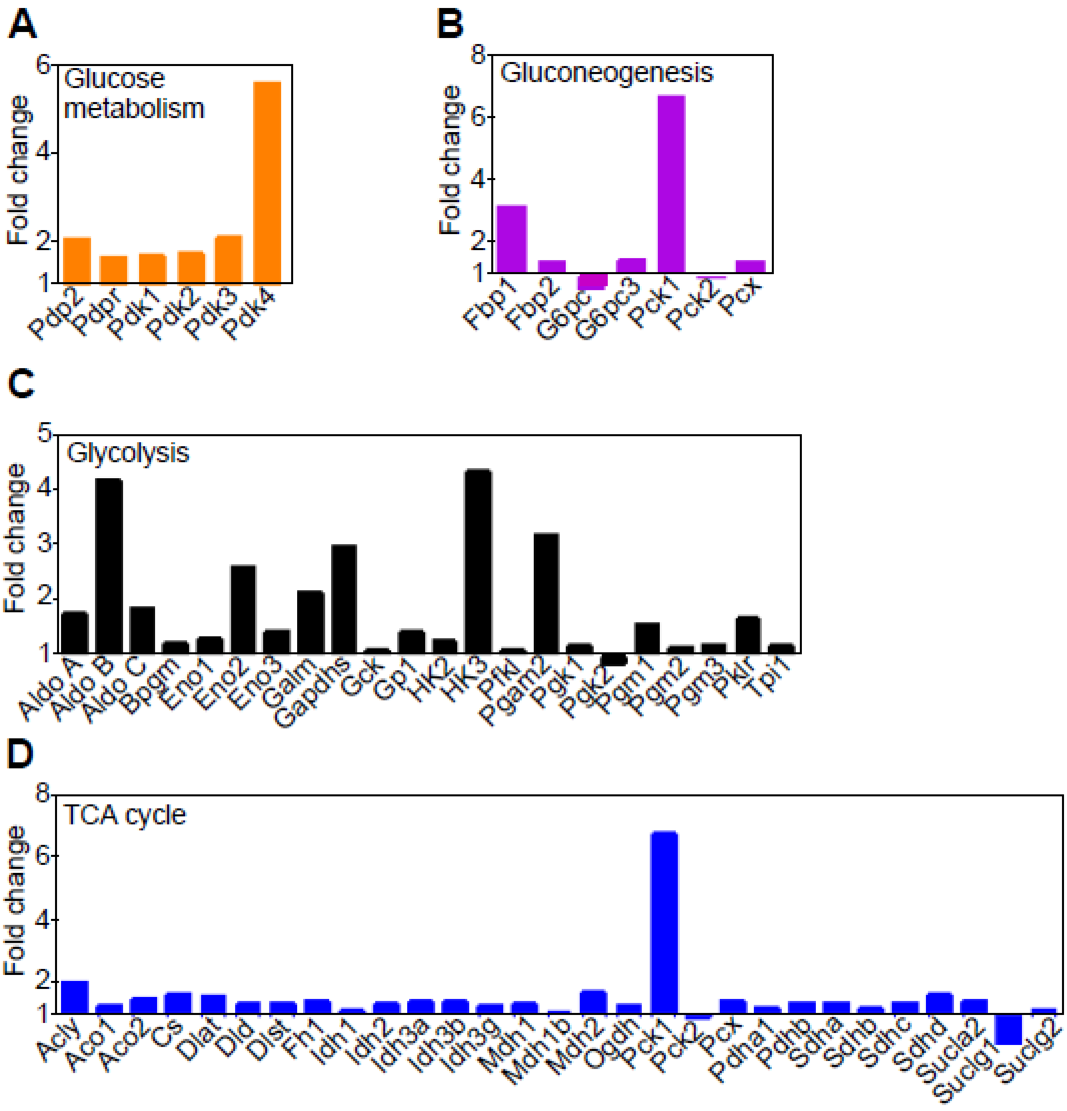

Supplementary figure 1

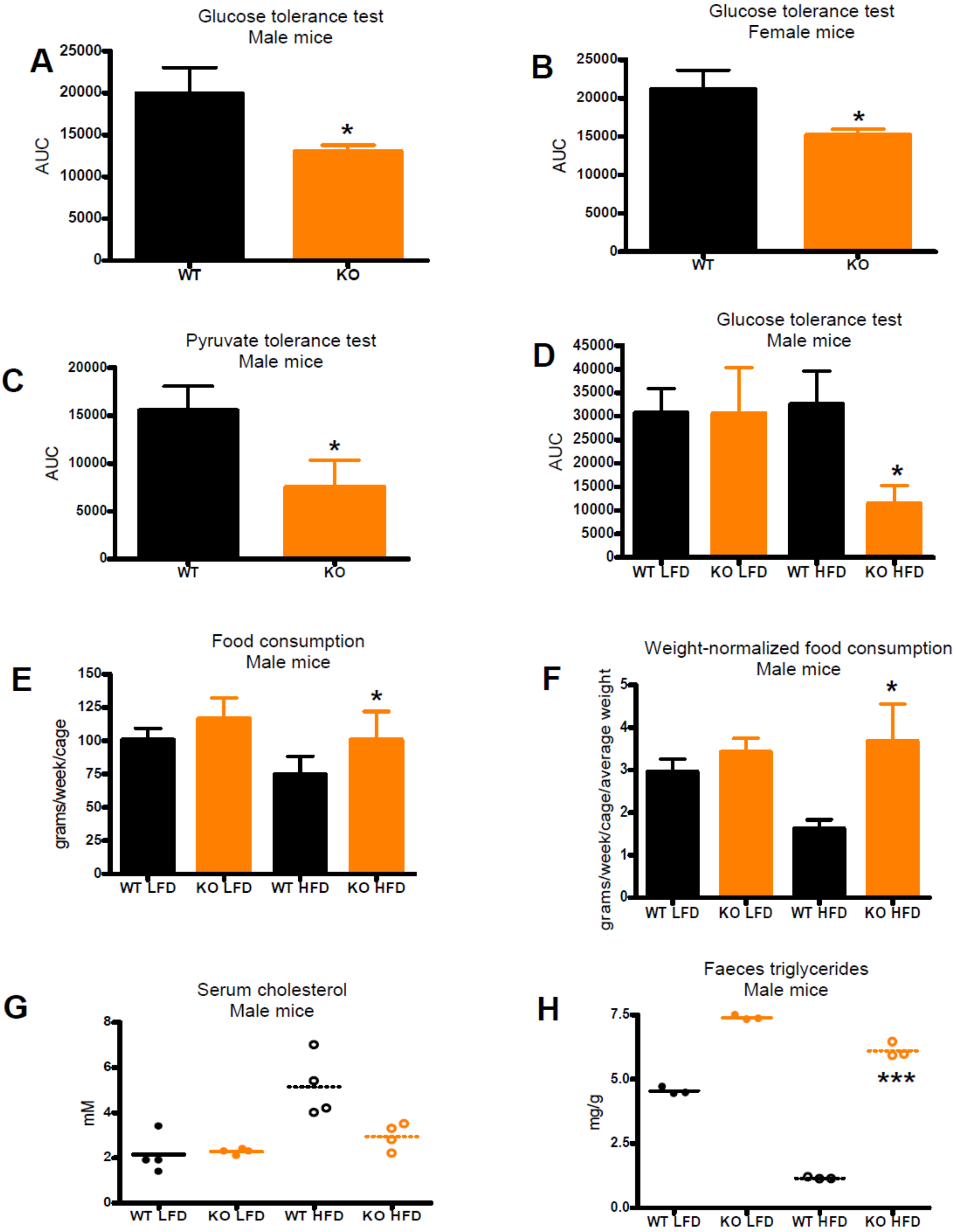

Supplementary figure 2

Animal ID	Body weight (g)	Liver weight (g)	Liver	Pancreas	Mesentery
699 KO LFD	28.2	1.3	0	0	0
2283 KO LFD	29	1.4	0	0	0
697 WT LFD	32.1	1.5	Moderate to severe multifocal to midzonal hepatic lipidosis (macro- and microvesicular) (3)	0	0
2275 WT LFD	35	1.73	Moderate to severe midzonal to diffuse hepatic lipidosis (macro- and microvesicular) (3)	0	0
698 KO LFD	28.9	1.4	Mild multifocal hepatic lipidosis (microvesicular) (1)	0	0
2773 WT LFD	30.5	1.4	Moderate diffuse hepatic lipidosis (microvesicular) (2)	0	0
2285 KO HFD	50.8	1.8	Moderate centrilobular to midzonal hepatic lipidosis (macro- and microvesicular) (2)	0	0
700 KO HFD	35.27	1.33	Mild multifocal hepatic lipidosis (macrovesicular) (1)	0	0
2290 WT HFD	55	4.03	Severe diffuse to centrilobular to midzonal hepatic lipidosis (macro- and microvesicular) (3)	large islet cells; Focal pyogranuloma (duct rupture, presumptive) with adjacent acinar loss and atrophy with acinar to ductal metaplasia	Mild multifocal fat necrosis with neutrophilic and histiocytic infiltrates (1)
2288 WT HFD	53.06	3.44	Severe diffuse to centrilobular to midzonal hepatic lipidosis (macro- and microvesicular) (3)	Focal pyogranuloma (duct rupture) with multinucleated giant cells and adjacent acinar loss and atrophy with acinar to ductal metaplasia	Mild multifocal fat necrosis with neutrophilic and histiocytic infiltrates (1)

WNL = within normal limits
*All have mild to moderate glycogen accumulation in the liver.
2290 - the islet cells appear noticably larger then in any other mice.

Scale:

0	WNL
1	Mild
2	Moderate
3	Severe

Supplementary figure 3

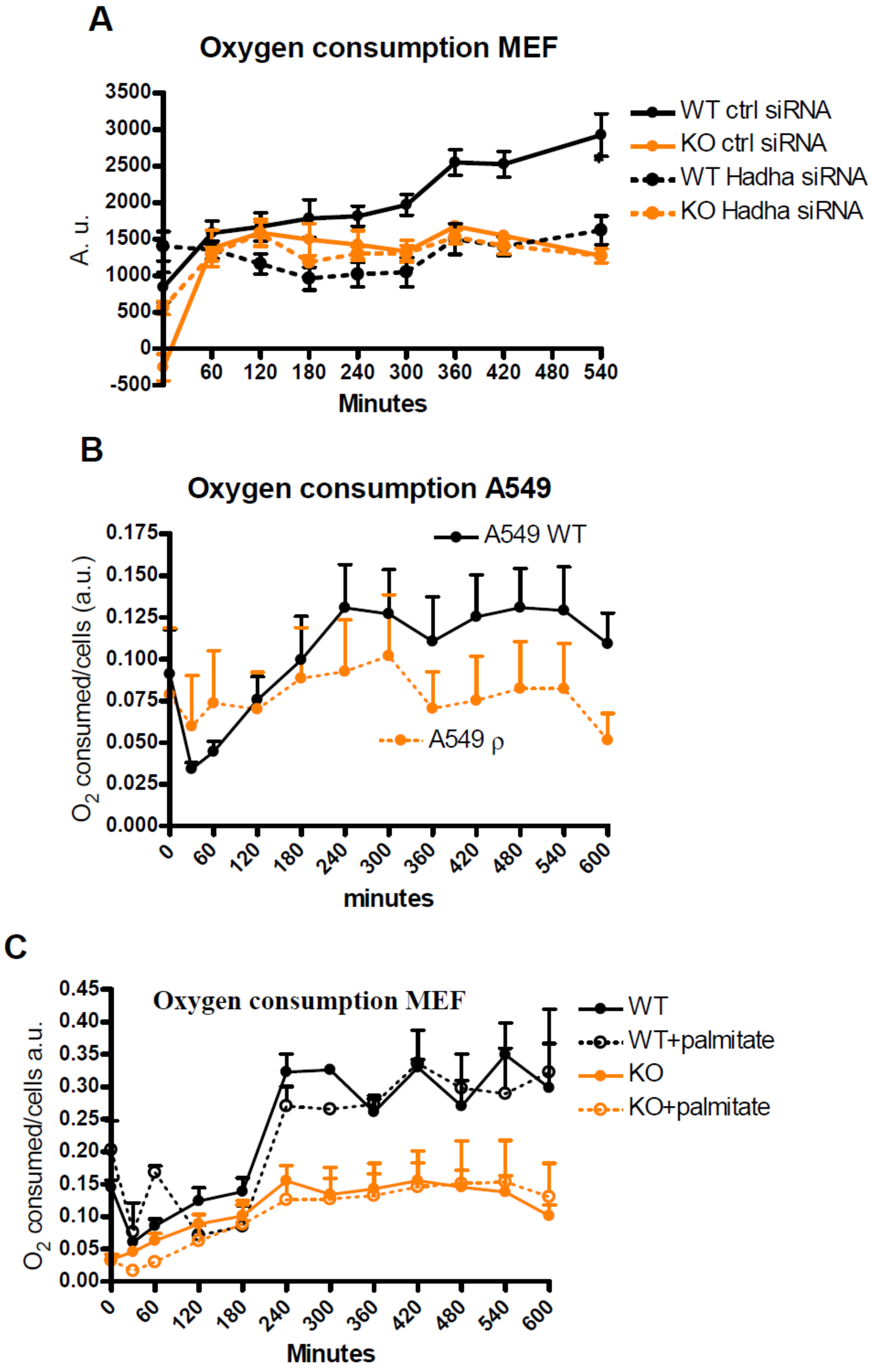

Supplementary figure 4

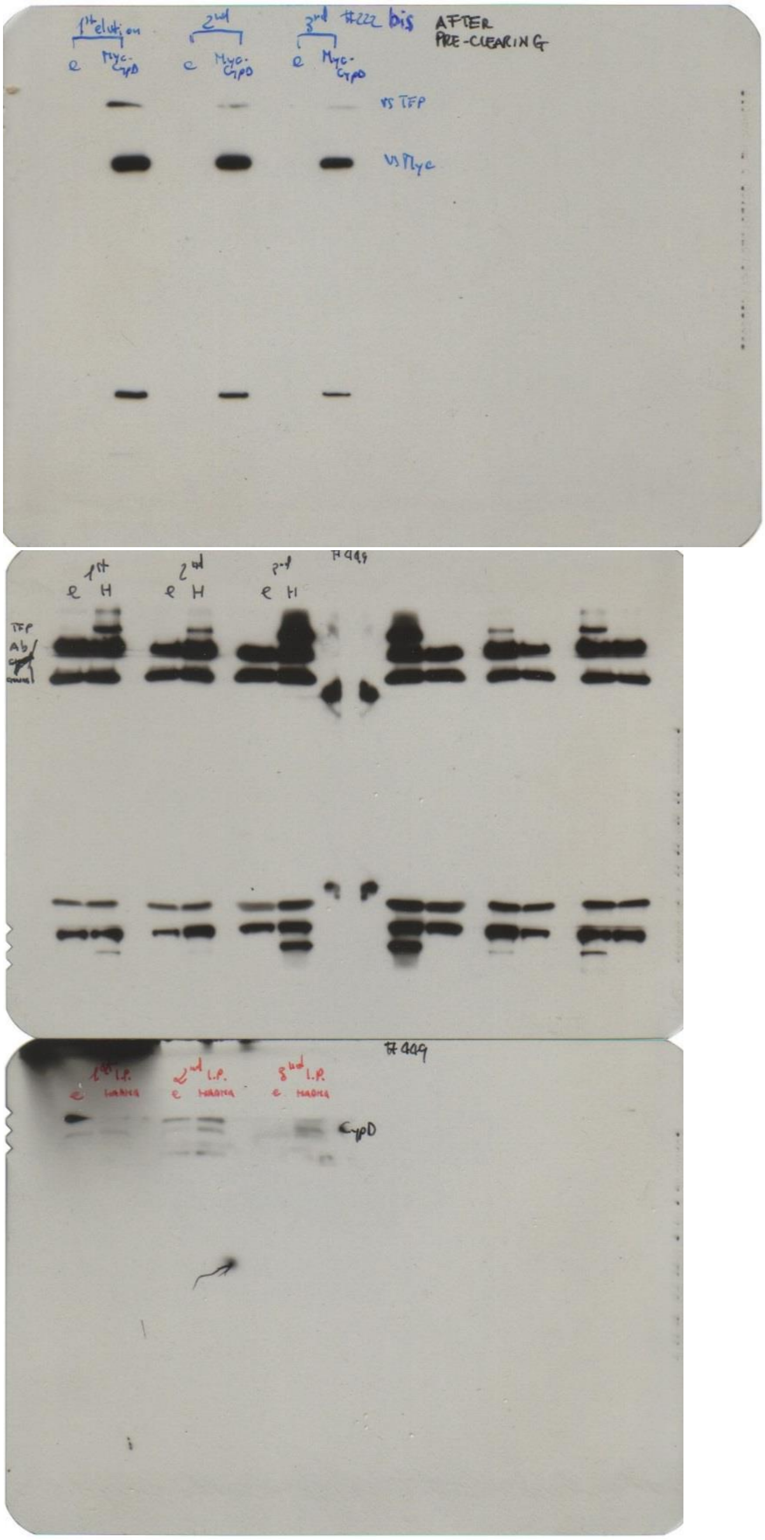

Supplementary figure 5

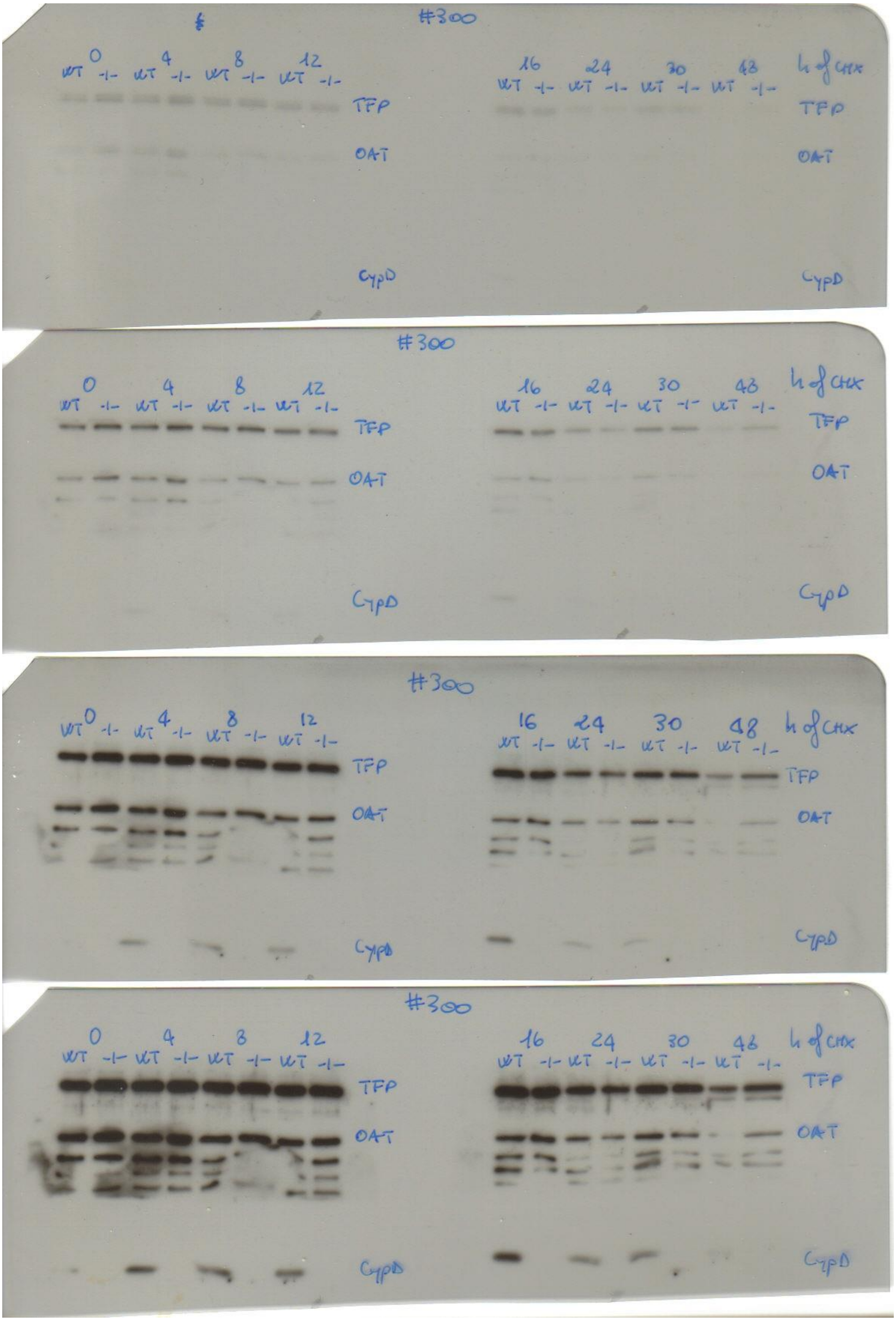

Supplementary figure 6

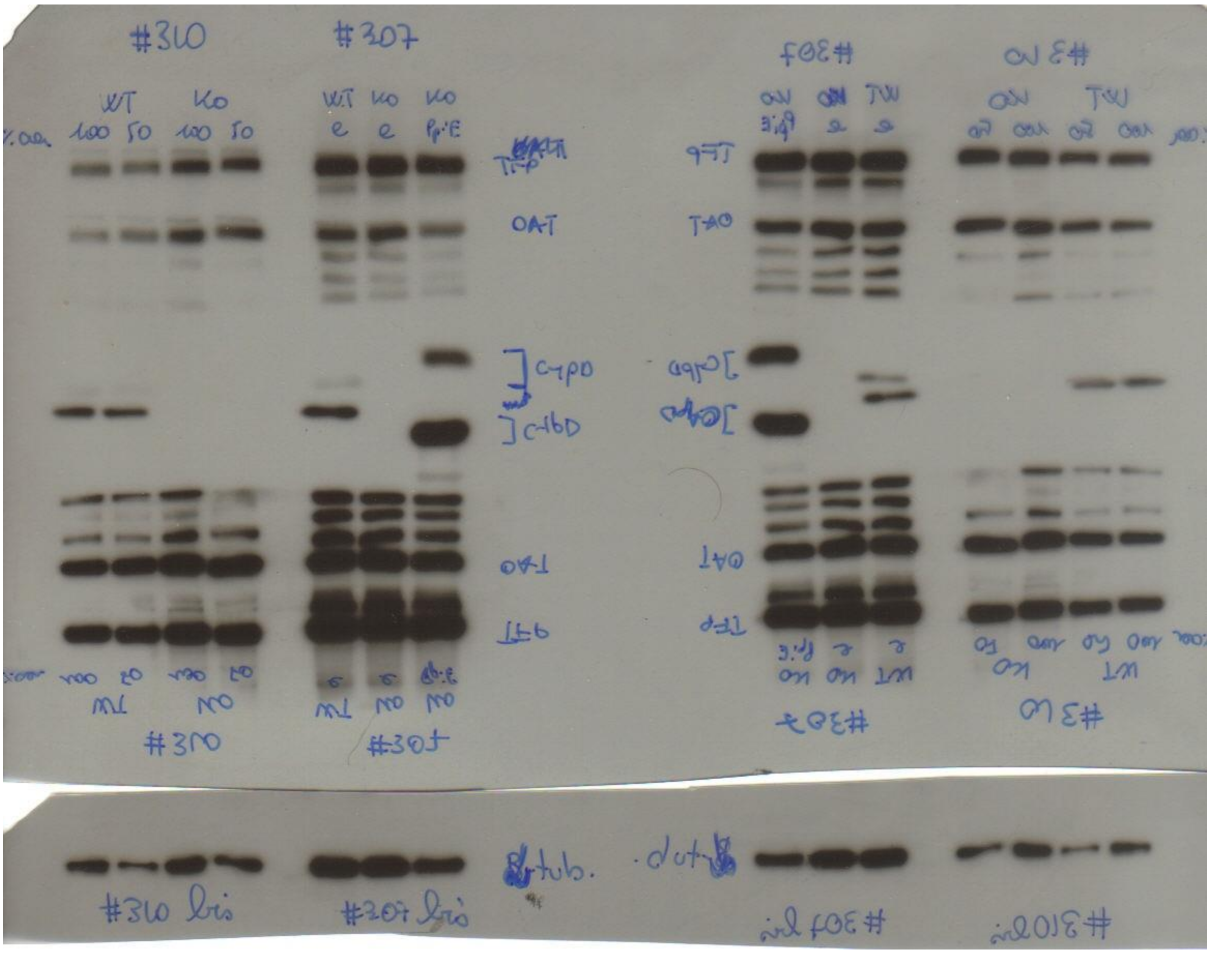

Supplementary figure 7

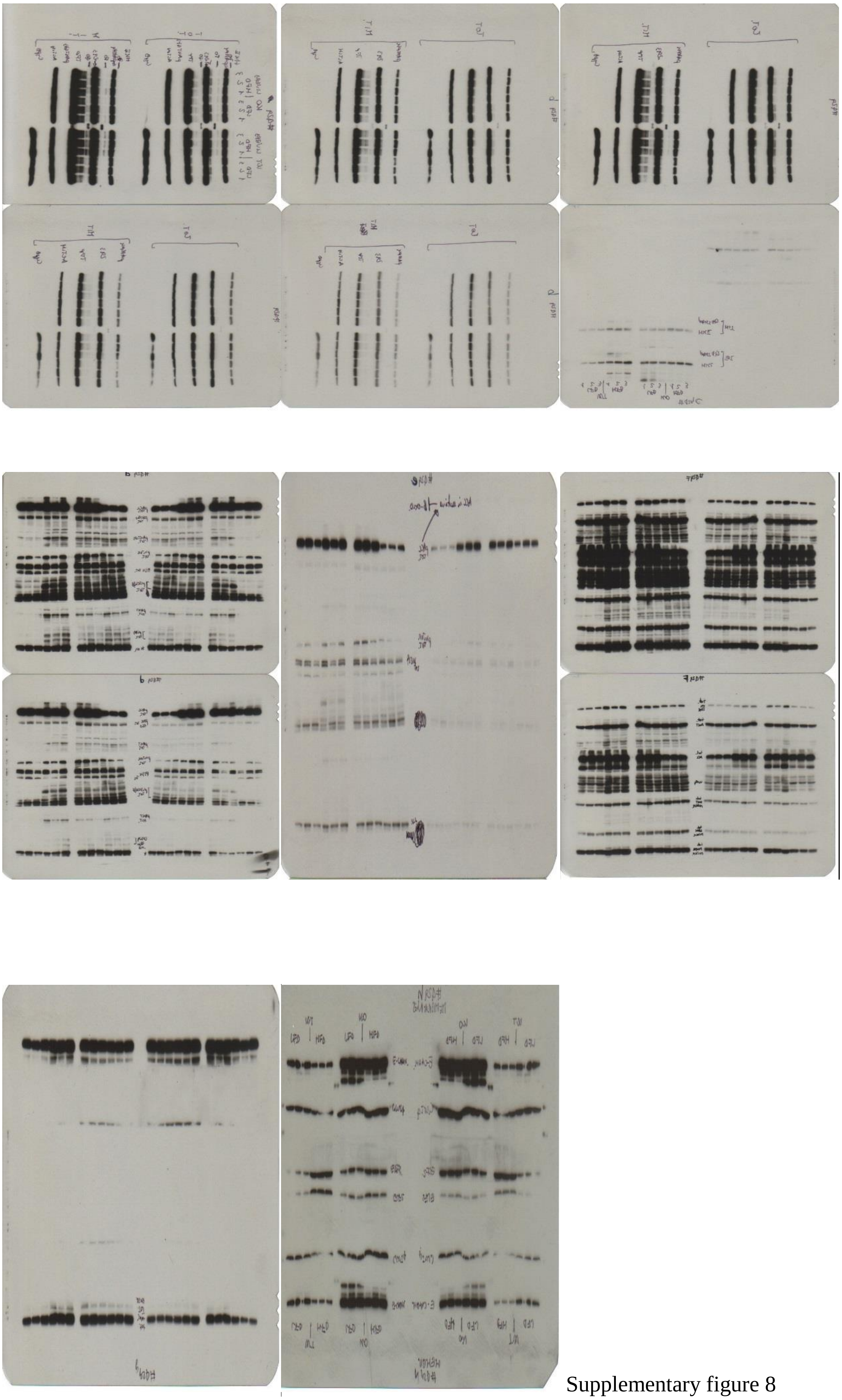

Supplementary figure 8

Supplementary Figure 1. **Gene expression profile of KO vs WT MEF.** Total RNA from WT or CypD KO MEF was extracted, reverse-transcribed and amplified for the indicated genes (glucose metabolism, **A**; gluconeogenesis, **B**; glycolysis, **C** and TCA cycle, **D**).

Supplementary Figure 2. **Area under the curve (AUC) of the GTT tests**. AUC were calculated for the data showed in figures 4 A,B,C and 5A (**A-D**), mean ± st. dev., n=4. Food consumption was measured every week for 12 weeks and showed as absolute consumption (**E**) or normalized on mice weight (**F**). Cholesterol (**G**) and triglycerides (**H**) were measured at the end of the 12-weeks diet period in serum and feces, respectively.

Supplementary Figure 3. **Pathological evaluations**. At the end of the 12 weeks diet period, mice were sacrificed and blindly evaluated and scored by an independent board-certified mouse pathologist.

Supplementary Figure 4. **Oxygen consumption.** Experiments were carried out as explained in the Materials and Methods section. (**A**) CypD WT and KO MEF were silenced for Hadha and their oxygen consumption analyzed. (**B**) WT and mtDNA-depleted (ρ0) A549 cells were analyzed for their oxygen consumption. (**C**) CypD WT and KO MEF were treated with palmitate and their oxygen consumption analyzed.

Supplementary Figure 5. **CypD/TFP interaction.** Films scans of western blot showing the interaction between CypD and TFP. Experiments were carried out as explained in the Materials and Methods section.

Supplementary Figure 6. **TFP stability.** Films scans of western blot showing the stability of TFP during CHX treatment. Experiments were carried out as explained in the Materials and Methods section.

Supplementary Figure 7. **CypD reconstitution experiment.** Films scans of western blot showing the reconstitution of CypD in KO MEF. Experiments were carried out as explained in the Materials and Methods section.

Supplementary Figure 8. ***In vivo* insulin pathway** Films scans of western blot showing the insulin pathway in livers from WT or KO animals after 12 weeks of diet. Experiments were carried out as explained in the Materials and Methods section.
